# Investigating Multi-cancer Biomarkers and Their Cross-predictability in the Expression Profiles of Multiple Cancer Types

**DOI:** 10.4137/bmi.s930

**Published:** 2009-05-01

**Authors:** George C. Tseng, Chunrong Cheng, Yan Ping Yu, Joel Nelson, George Michalopoulos, Jian-Hua Luo

**Affiliations:** 1 Department of Biostatistics, University of Pittsburgh, Pittsburgh, USA; 2 Department of Human Genetics, University of Pittsburgh, Pittsburgh, USA; 3 Department of Pathology, University of Pittsburgh, Pittsburgh, USA

**Keywords:** expression profile, meta-analysis, common signature, multi-cancer biomarker, carcinogenesis

## Abstract

Microarray technology has been widely applied to the analysis of many malignancies, however, integrative analyses across multiple studies are rarely investigated. In this study we performed a meta-analysis on the expression profiles of four published studies analyzing organ donor, benign tissues adjacent to tumor and tumor tissues from liver, prostate, lung and bladder samples. We identified 99 distinct multi-cancer biomarkers in the comparison of all three tissues in liver and prostate and 44 in the comparison of normal versus tumor in liver, prostate and lung. The bladder samples appeared to have a different list of biomarkers from the other three cancer types. The identified multi-cancer biomarkers achieved high accuracy similar to using whole genome in the within-cancer-type prediction. They also performed superior than the one using whole genome in inter-cancer-type prediction. To test the validity of the multi-cancer biomarkers, 23 independent prostate cancer samples were evaluated and 96% accuracy was achieved in inter-study prediction from the original prostate, liver and lung cancer data sets respectively. The result suggests that the compact lists of multi-cancer biomarkers are important in cancer development and represent the common signatures of malignancies of multiple cancer types. Pathway analysis revealed important tumorogenesis functional categories.

## Introduction

Human malignancies remain one of the leading causes of mortality in the United States. Uncontrolled growth, reduced ability to undergo apoptosis and the ability to metastasize are some of the important features of malignancies, regardless of origins of tissues. There are multiple mechanisms underlying the phenotype of cancer. The alterations of cell growth and cell death signaling pathway due to mutation and inactivation of tumor suppressor genes and/or amplification and activation of proto-oncogenes have been thought to be the primary causes of carcinogenesis.^1^ Abnormalities of the same signaling pathways can be found in multiple types of human cancers, while a tumor may contain multiple abnormalities in signaling. Overlapping these abnormalities among multiple types of tumors may shed light on some key alterations of carcinogenesis.

Prostate cancer is second only to skin cancer as the most commonly diagnosed malignancy in American men: at current rates of diagnosis, one man in six will be diagnosed with the disease during his lifetime.^2^ Even though nutritional and environmental etiology has been implicated for prostate cancer development, such link has yet to be firmly established in general population. Some studies suggested that up to 80% of men age older than 80 were found to contain pathologically recognizable prostate cancer, while rarely any man younger than 40 developed the same disease. This argues against any singular specific etiology responsible for prostate cancer besides aging. Histologically, prostate cancer cells closely interact with their neighbor stromal cells to form some distinctive architectural patterns that make up the basis of Gleason’s grading.^3^ The clinical courses of most prostate cancers are long, and some are life-threatening. Hepatocellular carcinoma, on the other hand, is quite the opposite. It is not age related, and is tightly linked to cancer etiologies such as alcohol, hepatitis B or C virus or certain toxins. Hepatocellular carcinoma is distinctive in its well confined nodular architecture. The clinical courses of most of the hepatocellular carcinomas are short and the fatality is high. Most of the lung cancers, with the exception of small cell carcinoma, are also associated with distinctive etiologies, such as smoking or chronic exposure to certain type of carcinogens. The urothelial carcinoma of the urinary bladder, however, is primarily idiopathic or viral related. Since these four types of cancer are so far apart in etiology, morphology and clinical courses, any common ground between these tumors could be interpreted as a likely common pathway of carcinogenesis.

In the literature, microarray technology has been widely applied to the analysis of many malignancies, including the four cancer types mentioned above. However, meta-analysis to integrate multiple studies has rarely been investigated. Segel et al.^4^ proposed a systematic approach to incorporate 1,975 arrays in 22 tumor types and constructed a large gene module map. The resulting module map was, however, too complex to follow up and the modules were based on 2,849 known biologically meaningful gene sets instead of learning new sets of multi-cancer biomarkers. The gene matching of heterogeneous array types also potentially deteriorate the analysis accuracy. In this report, we performed a meta-analysis on 455 arrays collected from four microarray studies in Affymetrix U95Av2 platform: 94 samples of liver tissue^5^ (43 liver cancer, 30 hepatic tissues adjacent to liver cancer, 21 normal liver from organ donors), 148 samples of prostate tissues^6^ (66 prostate cancer, 59 prostate tissues adjacent to prostate cancer and 23 organ donors), 151 samples of lung tissues^7^ (134 tumors and 17 normal lung tissues) and 62 urinary bladder tissues^8^ (5 normal and 57 tumors). The use of common array platform has avoided the problem of incorrect gene matching and gene annotation, a common cause to deteriorate the performance of meta-analysis in microarray.^9^ We performed two batches of analyses. In batch I, all three tissue types in liver and prostate were analyzed using analysis of variance (ANOVA) model. In batch II, normal and tumor tissues in all four cancer types were included and t-test was used to identify multi-cancer biomarkers (see Table 1 for data description). The identified biomarkers were found to have high predictability in both within-cancer-type (i.e. cross-validation within a single cancer type) and inter-cancer-type (i.e. prediction model trained in one cancer type and used to predict another cancer type) prediction via leave-one-out cross validation. Further pathway enrichment analysis identified statistically significant function categories of the biomarkers. Validation of the 47 batch II multi-cancer biomarkers on an independent 23 prostate tissues yielded 96% accuracy in inter-study prediction from the original prostate, liver and lung cancer data sets respectively, showing the robustness of the multi-cancer biomarkers and their implications to common carcinogenesis of multiple cancer types.

## Materials and Methods

### Data and preprocessing

We collected four published microarray data sets^5^–^8^ to perform meta-analysis on prostate, liver, lung and bladder samples. A total of 455 U95Av2 arrays were analyzed (94 liver, 148 prostate, 151 lung and 62 bladder tissues) with each covering 12,625 genes and EST sequences. The common array platform eliminated technical difficulties including gene matching and inter-platform discrepancies. In liver and prostate data sets, three types of samples were collected: organ donor (N), normal tissues adjacent to tumor (A) and tumor tissues (T). In lung and bladder tissues, only organ donor and tumor tissues were available. We analyzed the data through two batches of analyses. In the first batch, both liver and prostate data sets with all three tissues were included. The expression patterns across the three types of samples were the major targets for investigation. In the second batch, data of all four organ types were included and only normal and tumor samples were compared. For details see Table 1.

The raw data (CEL files) were preprocessed in each cancer type separately using dChip software for array quality assessment, normalization, expression intensity extraction and log-transformation (base 2). Genes of low information content in each data set were filtered respectively and the union gene set of the four data sets was retrieved for further analysis. Specifically, in each data set, the top 50% genes with the largest average intensities were first selected. Among them the top 50% genes with the largest standard deviations were further identified, resulting in 25% genes (3,156 genes) selected in each data set. The union list of these most informative 25% genes in four data sets was used for subsequent downstream analysis (a total of 5,917 genes). The expression intensities in each sample column of each data set are standardized to have zero mean and unit variance so that data sets of different cancer types are comparable.

### Biomarker selection by ANOVA and t-test

**Figure 1:**
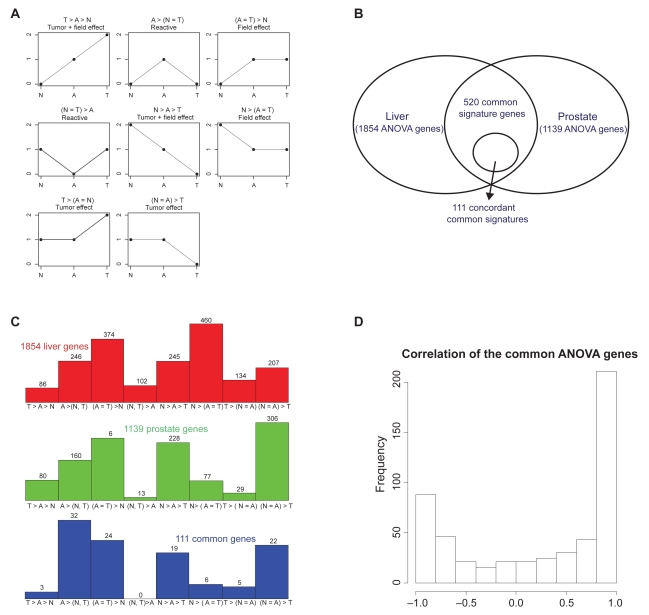
**ANOVA model for batch I analysis:** (**A**) Eight categories of ANOVA patterns used to select multi-cancer biomarkers. N denotes normal, A tissue adjacent to cancer, and T tumor sample. (**B**) Venn diagram representation of the number of ANOVA genes found to be significantly altered in liver and prostate tissues when comparing N, A and T groups. (**C**) Bar graph of genes that were altered in liver (1854), prostate (1139) or both tissue samples with same pattern (111). (**D**) Histogram of correlations of N-A-T patterns across prostate and liver of the 520 common ANOVA genes.

In batch I analysis, ANOVA model was fitted for the organ donor (N), adjacent to tumor (A) and tumor (T) samples with a β parameter for field effect and a γ parameter for tumor effect. Stepwise algorithm was used to select the best regression model. The ANOVA model is described in the following:

$$Y_{in}=\alpha_{i}+\beta_{i}\cdot F_{in}+\gamma_{i}\cdot T_{in}+{ɛ}_{in}$$

where *i* = 1, … 5917 for all the genes, *n* = 1, … 94 for liver samples and *n* = 1, … 148 for prostate samples. The field effect binary covariate *F_in_* = 1 for A or T group; *F_in_* = 0 for N group. The tumor effect covariate *T_in_* = 1 for T group; *T_in_* = 0 for N or T group. Field effect is defined as the expression difference between normal tissues (N) compared to tissues adjacent to tumor (A) and tumor tissue (T). Tumor effect is defined as a further difference between A and T. Genes satisfying the following criteria were selected: (a) statistical significance: adjusted q-value for the final stepwise-selected ANOVA model after Benjamini-Hochberg correction is less than 0.05 (i.e. to control false discovery rate smaller than 0.05); (b) biological significance: field effect or tumor effect is larger than 0.4 (correspond to ~32% fold change). The field effect and tumor effect parameter *β* and *γ* both have three possibilities- positive, negative and no change -, resulting in eight patterns as described in Figure 1A. Figures 1B and 1C show the number of genes selected in liver and prostate samples respectively and their distribution in the eight pattern categories. The intersection of selected ANOVA genes in liver and prostate with concordant pattern categories were used to construct prediction model for within-cancer-type (Liv→Liv and Pro→Pro) and inter-cancer-type (Liv→Pro and Pro→Liv) analysis. To summarize a list of gene markers in batch I for further analysis, genes selected in more than 70% of the times in leave-one-out cross validation (see section below for more detail) in the above procedure were identified as the “batch I multi-cancer biomarkers” (batchI-MBs).

In the batch II analysis, similar gene selection procedure was performed. Instead of ANOVA, simple t-test was performed to distinguish normal and tumor. Given the comparison of a pair of cancer types (e.g. liver vs. lung), genes satisfying the two criteria used in batch I were first selected and the intersection of the gene lists obtained from the two compared cancer types were identified. Among them, genes with concordant differential expression direction (up- or down-regulation) were used to construct prediction model for within-cancer-type (Liv→Liv and Lun→Lun) and inter-cancer-type (Liv→Lun and Lun→Liv) analysis. Leave-one-out cross validation was similarly performed. For each pair of cancer type comparison, gene lists of more than 70% appearance in the leave-one-out cross validation signatures were identified and were denoted as “liv-pro-MBs” (i.e. multi-cancer biomarkers in liver-prostate comparison), “liv-lun-MBs” etc. The intersection genes of “liv-pro-MBs”, “liv-lun-MBs” and “pro-lun-MBs” are denoted as “batchII-MBs” (See Fig. 4; bladder cancer data appear to generate a very different biomarker list than that from liver, prostate and lung data, as will be describe later).

### Gene-specific scaling in inter-cancer-type classification

**Figure 2:**
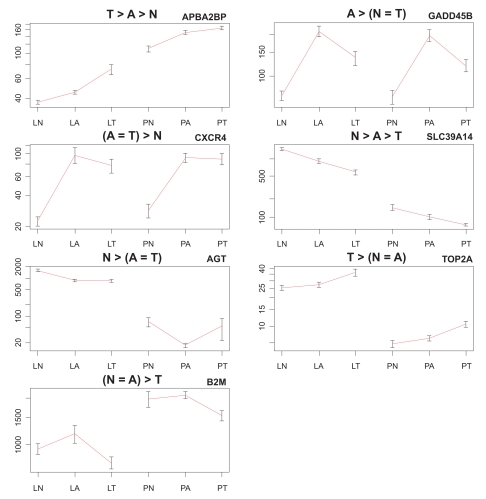
**Expression patterns of selected representative genes in liver and prostate samples.** Selected genes of seven pattern categories from the 111 common concordant ANOVA genes in liver and prostate samples. Global sample normalization has been performed across prostate and liver data sets. It is clearly seen that although all these biomarkers demonstrate concordant patterns across prostate and liver, many of them (APBA2BP, SLC39A14, AGT, TOP2A and B2M) are at different expression level and direct application of a prediction model developed in one data set will likely perform poor in the other data set.

Figure 2 demonstrates expression patterns of one selected gene for each of the eight pattern categories (the category (N = T) > A had no gene and is omitted). We observed that gene-specific scaling was needed for many of the biomarkers so the prediction information could be carried across organs. For example in “APBA2BP”, the expression of group A is consistently greater than N and group T is further greater than A in both liver and prostate samples. However, the levels of expression intensities in liver and prostate are in different scale even though all the liver and prostate samples are preprocessed and properly normalized across data sets. This phenomenon may be due to differential sample preparation, tissue physiology and/or hybridization conditions in different studies. As a result, we conducted gene-specific scaling in all inter-cancer-type classification. Conceptually the scaling parameters are estimated so that the gene vectors in each study are standardized to mean 0 and standard deviation 1. However, since each study has a different ratio of normal versus tumor samples, we performed a bootstrap sampling before scaling so that the gene vectors were standardized under a synthetic condition that groups (N, A and T) are of equal sample size in each study (see Appendix for more details).

### Classification method and leave-one-out cross validation

**Figure 3:**
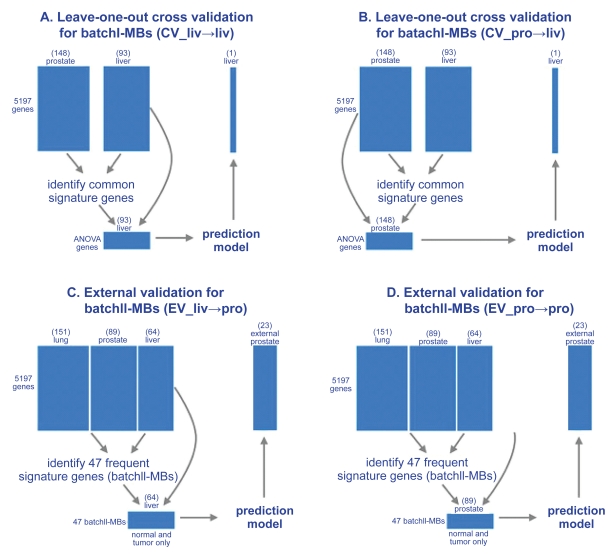
**Schemes of leave-one-out cross validation or external validation for batchI-MBs and batchII-MBs.** Upper: scheme for leave-one-out cross validation to evaluate the procedure of selecting batchI-MBs and batchII-MBs. The test sample is first left aside. The remaining samples are used for selecting multi-cancer biomarkers and constructing the prediction model to be used to evaluate the set-aside test sample. This scheme is used to evaluate procedures of selecting both batchI-MBs and batchII-MBs to generate Table 2 and Table 3. (**A**) an example to evaluate liv→liv in Table 2 (**B**) an example to evaluate pro→liv in Table 2. Lower: scheme for external validation of batchII-MBs by 23 independent prostate cancer samples. (**C**) external evaluation of the prediction model based on liver data and batchII-MBs (EV_liv→ pro). (**D**) external evaluation of the prediction model based on the old prostate data and batchII-MBs (EV_pro→pro).

PAM (Prediction Analysis of Microarray) was used to construct the prediction models in this paper.^10^ The method has been found effective in many microarray prediction analyses and has the merit that gene selection is embedded in the method. When “all genes” are used, the predictive genes are automatically chosen from the total of 5,917 genes to construct the prediction model. When “common signatures” are used, the common biomarkers are selected according to the description in the section “Biomarker selection by ANOVA and t-test” and no gene selection is further performed in PAM. Results of both gene selection procedures are reported and compared. To avoid over-fitting in the evaluation of cross-predictability of the multi-cancer biomarkers, we conducted rigorous leave-one-out cross validation (see the prediction scheme in Figs. 3A and 3B), i.e. the left out sample does not participate in the selection of marker genes.

### Confusion matrix and prediction index

In the literature, the overall accuracies from different methods are usually reported to compare performance. It is, however, often a misleading index in practice. Supplementary Table 1 demonstrates an example. Among 42 tumor patients, one false negative was made and among six normal patients, five false positives were made. The overall accuracy is pretty high (87.5%) but it is a result of predicting almost all tissues as tumor with high sensitivity (97.6%) but extremely low specificity (16.7%). A standard alternative to this situation may be the AUC (area under ROC curve) index by varying classification threshold in the classification rule. This measure is, however, not readily available for classical methods like KNN and SVM. Even for methods that can calculate AUC, the measure is very unstable for small sample size. In this paper, we report the confusion matrixes that convey the entire prediction results in the appendix. A 2 × 2 table is used to summarize the number of patients in true and predicted status of normal or tumor groups. The two off-diagonal numbers represent the false positives and false negatives in the prediction and their sum represent to total errors made (see Supplement Table 1). We then further summarize the prediction results by a prediction performance index (PPI) that is defined as the average of sensitivity and specificity, to be used throughout this paper for performance evaluation.

### Pathway analysis

For each gene list of multi-cancer biomarkers, the gene ontology (GO) database was used for pathway enrichment analysis. For each GO term, a Fisher’s exact test was performed to determine the enrichment of the gene list and a p-value was generated.^11^ We performed this analysis in batchI-MBs, batchII-MBs and all pairwise comparison multi-cancer biomarkers in batch II (liv-pro-MBs, liv-lun-pro-MBs etc). The p-value results were summarized in a heatmap (Fig. 5).

### External evaluation of batchII-MBs by independent prostate data

A data set of 23 prostate cancer samples performed in an independent lab^12^ was used for external validation of the batchII-MBs. A toltal of 47 batchII-MBs were identified from the normal and tumor samples in liver, prostate and lung data sets. To evaluate the robustness and inter-cancer-type cross-predictability, a prediction model based on the 47 batchII-MBs in the normal and tumor samples of liver data set was constructed and was used to evaluate the 23 external prostate cancer samples (see “EV_liv→pro in Fig. 3C). The evaluation of prediction model generated by the old prostate data is denoted by “EV_pro→pro” in Figure 3D. Similarly we also perform “EV_lun→pro” evaluation. The data preprocessing of the 23 new samples was conducted similarly to the four analyzed data sets and simple constant normalization was adopted against the original prostate data set. Additional gene-wise normalization against the original prostate is also applied so the liver and lung data sets can be used to predict the 23 new prostate samples.

## Results

To identify common signature genes among four types of malignancies, we started with the prostate and liver data sets in batch I analysis because of more balanced numbers of tumor and normal samples and availability of benign tissues adjacent to tumor. In this analysis, 1,854 genes from liver data set and 1,139 genes from the prostate data set were found to fit the ANOVA model and meet the gene selection criteria. Among these genes, 520 genes were common in both organs (Venn diagram in Fig. 1B). The histogram of correlations of N vs A vs T patterns (average intensities of each group) across two organs in each gene is shown in Figure 1D. Majority of the genes were highly correlated across prostate and liver but surprisingly 113 genes presented strong negative correlation (<−0.7), which may reflect the differences in tissue types. The 520 selected genes were categorized into eight patterns as demonstrated in Figure 1A. These patterns represent either tumor specific alteration, field effect, or reactive changes. Among these 520 genes, 111 genes were in the same pattern categories in liver and prostate (Fig. 1C) based on our definition in Figure 1A. Further analysis of expression of the 111 genes in both organs indicated that even though the expression patterns for these genes across N, A and T were identical in both organs, the levels of expressions may vary greatly (for example, APBA2BP and SLC39A14 in Fig. 2). This suggests that direct application of classification model constructed in one cancer type may not predict the histology of tissues in the other cancer type. To resolve this problem, an adequate gene-specific scaling across organs was carried out for the inter-cancer-type prediction. The gene-specific scaling procedure described in the Method section and Appendix is applied for all analyses hereafter.

We performed leave-one-out cross validation throughout the prediction analyses. There are 242 samples in liver and prostate data sets. Among the 242 leave-one-out cross validation analysis, a total of 109 common biomarkers were identified in more than 70% leave-one-out cross validation and all of them belong to the 111 gene list using all liver and prostate samples described above. These 109 frequently identified biomarkers are named “batchI-MBs”. 99 (out of 109) were identified as distinct multi-cancer biomarkers (Supplement Table 4). Subsequently we assessed the cross-predictability of the identified biomarkers. When using all genes, we observed high PPI between normal and tumor comparison (N vs. T) with 96.5% in liver dataset and 93.9% prostate dataset while lower accuracy was observed between adjacent and tumor (79.9% in liver and 71.4% in prostate) (Table 2). When only common signature biomarkers were used, the prediction accuracy remained comparable to using all genes (N vs. T: 96.5% in liver and 98.8% in prostate; A vs. T: 75.6% in liver and 66.7% in prostate). The result suggests that the common signature biomarkers carry as good predictive information as the entire 5,917 genes. We then further conducted inter-cancer-type classification analysis. We used either “all genes” (the entire 5,917 genes) or the common signatures to construct a prediction model in one cancer type and predict in another cancer type. The prediction evaluation was performed in a manner of leave-one-out cross validation. We denoted “prostate→liver” as constructing prediction models using prostate samples and predicting liver samples. We found that prediction with “all genes” did not perform well with only 47.4% in liver→prostate and 66.3% in prostate→liver among N vs T comparison and 55.7% in liver→prostate and 51.9% in prostate→liver among A vs T comparison. On the other hand, the model using common signature genes achieved much superior performance, nearly as good as the within-cancer-type classification (96.3% in liver→prostate and 93% in prostate→ liver among N vs. T comparison and 65.1% in liver→prostate and 74.7% in prostate→liver among A vs. T comparison). The results clearly demonstrate the cross-predictability of the common signatures.

**Figure 4:**
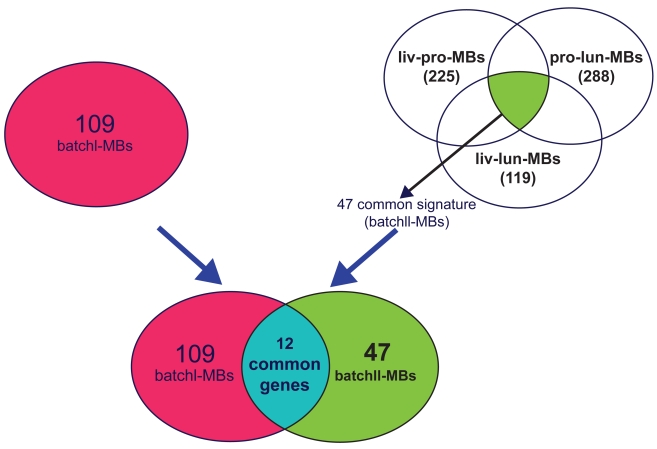
Diagram of batchI-MBs and batchII-MBs and their intersection genes. The 47 batchII-MBs are listed in Table 5 and 109 batchII-MBs are listed in Supplement Table 4.

**Figure 5:**
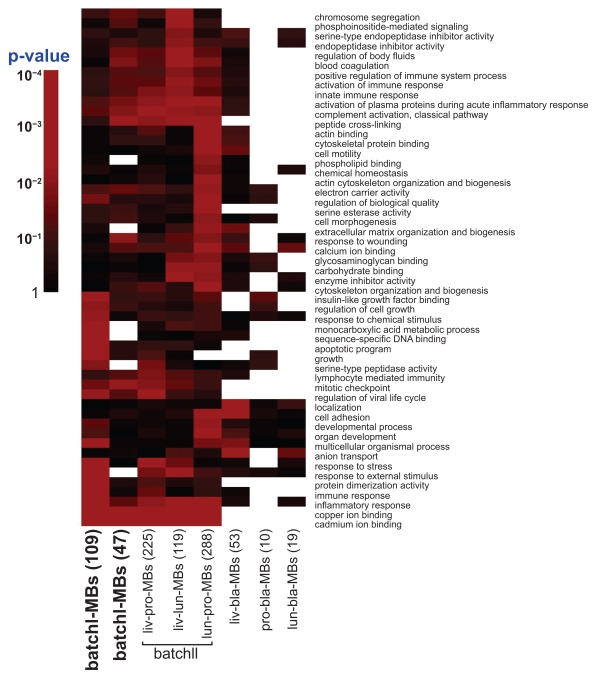
**Pathway analysis heatmap.** The enriched Gene Ontology terms are demonstrated on rows and lists of multi-cancer biomarkers are shown on columns. The significance (p-values) is represented by gradient red color. When the number of genes of the biomarker list that fall in the GO term is too small or zero, the p-value assessment is not computable or not stable and is represented as missing in white color.

Subsequently, we expanded our analysis to prostate, lung, liver and bladder data sets (batch II analysis) with only normal and tumor tissues to test whether common signature genes can be found across these four types of cancers. Similar analyses were performed except that ANOVA was replaced by t-test for two class normal and tumor comparison. Each pair of the cancer types was analyzed. Similar to batch I analysis, only common signature genes with consistent regulation direction (up-regulation or down-regulation) in both cancer types were selected. Table 3 (see also Supplement Table 3 for the entire confusion matrix results) summarizes the prediction results of batch II analysis. Similar to the result of batch I analysis, we observed high prediction accuracy for within-cancer-type prediction when using all genes in PAM (96.5% for liver, 93.9% for prostate, 90.7% for lung and 88.6% for bladder). The prediction models using common signature biomarkers generated similar high accuracy compared to using all genes (91.7%–97.7% in liver, 79.6%–95.6% in prostate, 89.4%–96.0% in lung and 97.4%–98.3% in bladder). The result confirms that the common signature biomarkers carry as good predictive information as the entire 5,917 genes. For the inter-cancer-type classification analysis, we repeatedly found that prediction with all genes did not perform well. In contrast, using common signature genes achieved much superior performance (Table 4). Liver particularly seemed to be the most robust either used as training or test data. Bladder, however, showed slightly lower cross-predictability with the other three cancer types. The numbers of common signature genes of bladder with other cancer types are also much smaller. Following the same criterion of selecting 70% frequency of being selected as common signatures in the cross-validations, we identified multi-cancer biomarkers of the comparison in each pair of cancer types in Table 4 (255 liv-pro-MBs, 119 liv-lun-MBs, 288 lun-pro-MBs, 53 liv-bla-MBs, 10 pro-bla-MBs and 19 lun-bla-MBs). When all possible pairs of comparisons among liver, prostate and lung are overlapped (liv-pro-lun-MBs), a number of 47 genes was identified. After deleting replicates, 44 (out of 47) distinct multi-cancer biomarkers in liver, prostate and lung cancers were identified as batchII-MBs (Table 5). However, these common signature genes do not overlap with those from bladder data set, indicating a lack of common signature between these cancers and bladder cancer. There are 12 overlapping genes (Fig. 4; p < 1E-10 with significantly high overlapping) between batchI-MBs and batchII-MBs (marked with asterisk in Table 5 and Supplement Table 4). Pathway analysis was performed on these multi-cancer biomarkers indicating that fewer numbers of multi-cancer biomarkers and GO terms were identified when bladder samples were analyzed in the inter-cancer-type prediction.

**Figure 6:**
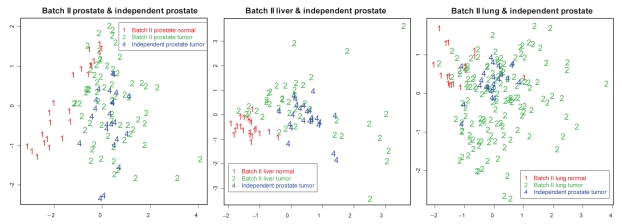
**MDS plot of existing training data set and independent prostate cancer data.** Three MDS plots of the existing liver, prostate and lung training data sets respectively with the 23 independent prostate tumor samples. The mixing of the 23 tumor samples and old tumor samples exclude the possibility of accidental high accuracy due to study differences.

To validate the robustness and cross-predictability of batchII-MBs, a data set of 23 independent prostate cancer samples obtained from another institute^12^ was evaluated. The prediction model based on the 47 batchII-MBs in the 64 normal and tumor liver samples achieved 96% (22/23) accuracy in predicting the 23 independent prostate samples (the “EV_liv→pro” scheme in Fig. 3C). Evaluation of “EV_pro→pro” and “EV_lun→pro” also gave the same results (96% accuracy). Since we only have tumor samples in the external prostate data, there is a potential pitfall that the high accuracy may be an accidental result of study discrepancies between the new 23 prostate samples and the normal and tumor samples in analyzed data sets. We performed multi-dimension scaling (MDS) plots to visualize the new and old samples and excluded this possibility (Fig. 6). The new prostate tumor samples are scattered and mixed with the old tumors but separated from old normal samples. As a result, the high accuracy of the prediction on this new data set is not caused by pure “accident.”

## Discussion

Meta-analyses have been performed for several types of human malignancies.^13^–^18^ However, to our knowledge, this is the first report showing that a microarray gene expression model demonstrates inter-cancer predictability between different types of cancers using the identified multi-cancer biomarkers. These results not only were evaluated in cross-validation analysis of existing working data sets but also were validated by independent prostate tissues collected and preprocessed separately. This argues strongly in favor of the reproducibility of the multi-cancer biomarkers and the models. The 44 batchII-MBs appear to represent the common gene expression alteration among hepatocellular carcinoma, lung and prostate cancer. They follow similar patterns of differential expression in normal and tumor tissues for prostate, lung and liver cancer. Surprisingly, these gene signatures predict prostate, lung and hepatocellular carcinoma with similarly high accuracy as using the entire genome information of 5917 genes in each within-cancer-type prediction in prostate, lung or liver cancer. This suggests that the 44 genes are the major determinant of gene expression alteration in these three types of cancers.

Comparing the 44 genes to published potential biomarker list yielded high overlapping (28 overlapped to the 3,312 gene list generated in Bhattacharjee et al.^7^ 22 overlapped to the 2,413 gene list generated in Luo et al.^5^ 16 overlapped to the 726 gene list generated in Yu et al.^6^). The high level of inter-organ cancer predictability using just 44 genes implies that the core of cancer gene alterations may actually be quite small. The alterations of the expression of these genes could represent the common features of the three types of malignancies. None of these genes was, however, identified as the most significantly altered in bladder cancer suggest the dis-resemblance of bladder cancer to these three types of cancers. Among these genes includes a interferon inducible protein, *1–8D* (IFITM2, 411_i_at). This gene was a known important mediator of inter-feron induced in cell growth inhibition and induction of cell death.^19^,^20^ 1–8D was down-regulated in hepatocellular carcinoma, lung cancer and prostate cancer, while pro-growth genes such as *cyclin B1* (CCNB1, 34736_at) was significantly up-regulated in three types of tumor samples. Other genes involving in growth controls including growth arrest specific 6 (GAS6, 1597_at), G0/G1swtich 2 (GOS2, 38326_at) are also abnormally expressed in these tumors. The 44 gene list also includes six metallothioneins including 1A, 1B, 1E, 1F, 1H and 2A (MT1A, 31623_f_at; MT1B, 609_f_at; MT1E, 36130_f_at; MT1F, 31622_f_at; MT1H, 39594_f_at; MT2A, 39081_at). Metallothioneins are some low molecular weight zinc binding proteins that play important role in regulating transcriptional activity for variety of genes, and play crucial role in zinc signaling.^21^,^22^. Abnormal up-regulation of these genes may result in global pattern of gene expression alteration. Up-regulation of metallothioneins were thought to contain prognostic value in invasive ductal breast cancer.^23^ CCNB1 and most of the metallothioneins were also identified in batchI-MBs where adjacent tissues were included in the analysis. In the pathway analysis, we also observe many cancer related functional categories, including “mitotic checkpoint”, “apoptotic program”, “copper ion binding” and “cadmium binding”. Investigation into the abnormalities of these pathways may yield important insight into the common carcinogenesis mechanism of the tumors. A possible future work is to study sequential biopsies in the progression of different tumors in a mouse model and analyze the expression changes of the biomarkers identified in this paper. Such rigorous validation of signature genes can help create a carcinogenic model and reduce the inter-individual genetic differences.

The clinical implication of our finding is two-fold: If the prediction of hepatocellular carcinoma, lung cancer and prostate cancer using our 44 batchI-MBs is interchangeable, we like to hypothesize that the abnormalities in the expression of the 44 genes represent a common features of these malignancies. Therapeutic targeting toward some of these genes will be of significant value in treating these malignancies. Second, the 99 batchII-MBs predicts tissues adjacent to malignancies versus completely normal organ tissues with high accuracy. This model may be able to serve as predictor of malignancies nearby even if a biopsy misses its tumor target. This may serve as an indicator for a quick follow-up re-biopsy until the tumor(s) is identified. Alternatively, the detection of a strong cancer field effect change may argue for some prophylactic treatments before morphological cancer appears.

## Supplementary Material

### Bootstrap procedure for gene-wise normalization

Conceptually we standardize each gene vector to mean 0 and standard deviation 1 to accommodate different expression range of a predictive biomarker across different studies (e.g. APBA2BP, SLC39A14, AGT, TOP2A and B2M in Fig. 2). Since the ratios of normal and tumor groups can vary in different studies, simple standardization can cause bias and deteriorate the prediction performance. Instead we perform bootstrap to sample a gene vector of B = 1,000 samples in each group and standardize the vector of 2,000 (3,000 if N, A and T groups are all compared) bootstrapped samples to mean 0 and standard deviation 1 to estimate the standardization factors. Essentially we perform standardization under the simulated condition that normal and tumor groups have the same sample sizes.

**Supplement Table 1:** An example of confusion matrix. Two false negatives and five false positive are made in the prediction, which sum up to seven total errors (with 42/48 = 87.5% overall accuracy). The sensitivity is 41/42 = 97.6%, specificity 1/6 = 16.7% and prediction performance index (PPI) (97.6% + 16.7%)/2 = 57.2%.

	True normal tissues	True tumor tissues
Predicted as normal tissues	1	1
Predicted as tumor tissues	5	41

**Supplement Table 2:** Batch I leave-one-out cross validation analysis result (confusion matrix).

			**Liver vs Prostate (Normal vs Tumor)**									
			**Liver → Liver**		**Prostate → Liver**				**Prostate → Prostate**		**Liver → Prostate**	
			**True N**	**True T**	**True N**	**True T**			**True N**	**True T**	**True N**	**True T**
**Liver vs Prostate (Normal vs Tumor)**												
All genes	69	Predicted N	21	3	21	29	55	Predicted N	23	8	19	58
		Predicted T	0	40	0	14		Predicted T	0	58	4	8
Common signature	111.3	Predicted N	21	3	20	4	111.9	Predicted N	23	1.6	22	2
		Predicted T	0	40	1	39		Predicted T	0	64.4	1	64
**Liver vs Prostate (Normal vs Adjacent)**												
			**Liver → Liver**		**Prostate → Liver**				**Prostate → Prostate**		**Liver → Prostate**	
			**True N**	**True A**	**True N**	**True A**			**True N**	**True A**	**True N**	**True A**

All genes	66	Predicted N	20	3	18	9	63	Predicted N	23	4	15	33
		Predicted A	1	27	3	21		Predicted A	0	55	8	26
Common signature	111.2	Predicted N	21	1.1	20	1	110.4	Predicted N	23	2	23	4
		Predicted A	0	28.9	1	29		Predicted A	0	57	0	55
**Liver vs Prostate (Adjacent vs Tumor)**												
			**Liver → Liver**		**Prostate → Liver**				**Prostate → Prostate**		**Liver → Prostate**	
			**True A**	**True T**	**True A**	**True T**			**True A**	**True T**	**True A**	**True T**

All genes	64	Predicted A	27	13	13	17	266	Predicted A	44	21	46	44
		Predicted T	3	30	17	26		Predicted T	15	45	13	22
Common signature	111.5	Predicted A	27	16.7	26	16	112.0	Predicted A	42	25	41	26
		Predicted T	3	26.3	4	27		Predicted T	17	41	18	40

* The numbers marked in dark gray are the number of genes used to construct the prediction model. When “all genes” are used, the PAM method performs automatic gene selection to construct the model. When “common signature genes” are used, no gene selection is performed in PAM and the results (number of genes and confusion matrix) shown are averages of leave-one-out cross-validation results.

**Supplement Table 3:** Batch II leave-one-out cross validation analysis result (confusion matrix).

			**Liver vs Prostate**									
			**Liver → Liver**		**Prostate → Liver**				**Prostate → Prostate**		**Liver → Prostate**	
			**True N**	**True T**	**True N**	**True T**			**True N**	**True T**	**True N**	**True T**

All genes	69	Predicted N	21	3	21	29	55	Predicted N	23	8	19	58
		Predicted T	0	40	0	14		Predicted T	0	58	4	8
Common signature	225.9	Predicted N	21	2	21	2	222.0	Predicted N	21.3	1	21	2
		Predicted T	0	41	0	41		Predicted T	1.7	65	2	64
**Liver vs Lung**												
			**Liver → Liver**		**Lung → Liver**				**Lung → Lung**		**Liver → Lung**	
			**True N**	**True T**	**True N**	**True T**			**True N**	**True T**	**True N**	**True T**

All genes	69	Predicted N	21	3	21	37	57	Predicted N	16	17	13	115
		Predicted T	0	40	0	6		Predicted T	1	117	4	19
Common signature	120.1	Predicted N	21	4.1	21	6	120.3	Predicted N	16	3	16	6
		Predicted T	0	38.9	0	37		Predicted T	1	131	1	128
**Lung vs Prostate**												
			**Lung → Lung**		**Prostate → Lung**				**Prostate → Prostate**		**Lung → Prostate**	
			**True N**	**True T**	**True N**	**True T**			**True N**	**True T**	**True N**	**True T**

All genes	57	Predicted N	16	17	17	83	55	Predicted N	23	8	23	49
		Predicted T	1	117	0	51		Predicted T	0	58	0	17
Common signature	289.5	Predicted N	16	6	16	7	288.8	Predicted N	17	9.7	15	13
		Predicted T	1	128	1	127		Predicted T	6	56.3	8	53
**Liver vs Bladder**												
			**Liver → Liver**		**Bladder → Liver**				**Bladder → Bladder**		**Liver → Bladder**	
			**True N**	**True T**	**True N**	**True T**			**True N**	**True T**	**True N**	**True T**

All genes	69	Predicted N	21	3	21	32	135	Predicted N	5	13	4	46
		Predicted T	0	40	0	11		Predicted T	0	44	1	11
Common signature	51.7	Predicted N	21	7.1	21	7	54.5	Predicted N	5	2	5	2
		Predicted T	0	35.9	0	36		Predicted T	0	55	0	55
**Prostate vs Bladder**												
			**Prostate → Prostate**		**Bladder → Prostate**				**Bladder → Bladder**		**Prostate → Bladder**	
			**True N**	**True T**	**True N**	**True T**			**True N**	**True T**	**True N**	**True T**

All genes	55	Predicted N	23	8	16	64	135	Predicted N	5	13	4	54
		Predicted T	0	58	7	2		Predicted T	0	44	1	3
Common signature	9.5	Predicted N	20.3	1.6	18	3	10.1	Predicted N	5	2.5	4	2
		Predicted T	2.7	64.4	5	63		Predicted T	0	54.5	1	55
**Lung vs Bladder**												
			**Lung → Lung**		**Bladder → lung**				**Bladder → Bladder**		**Lung → Bladder**
			**True N**	**True T**	**True N**	**True T**			**True N**	**True T**	**True N**	**True T**

All genes	57	Predicted N	16	17	17	129	135	Predicted N	5	13	5	56
		Predicted T	1	117	0	5		Predicted T	0	44	0	1
Common signature	19.1	Predicted N	14.9	11.9	15	22	19.2	Predicted N	5	3	4	5
		Predicted T	2.1	122.1	2	112		Predicted T	0	54	1	52

* The confusion matrixes in the gray shaded regions are used to generate the PPI in shaded regions in Table 3 and the corresponding Table 4.

**Supplement Table 4:** A total of 109 biomarkers are identified in more than 70% of leave-one-out cross validation in batch I (batchI-MBs). After deleting duplicates, 99 distinct predictive biomarkers are listed below.

Probe set ID	Gene title	Gene symbol	Signed mean fold Change			
			Liver		Prostate	
			A-N	T-N	A-N	T-N
39597_at*	actin binding LIM protein family, member 3	ABLIM3	−1.4	−2.1	−1.3	−1.6
37599_at*	aldehyde oxidase 1	AOX1	−1.5	−2.8	−1.6	−2.6
34736_at*	cyclin B1	CCNB1	1.3	2.3	1.2	1.8
37302_at*	centromere protein F, 350/400 ka (mitosin)	CENPF	1.3	1.9	1.1	1.4
37203_at*	carboxylesterase 1 (monocyte/macrophage serine esterase 1)	CES1	−1.3	−1.8	1	−1.7
32168_s_at*	Down syndrome critical region gene 1	DSCR1	1.1	−2	1.1	−1.6
34311_at*	glutaredoxin (thioltransferase)	GLRX	−1.6	−2.5	−1.3	−1.7
1737_s_at*	insulin-like growth factor binding protein 4	IGFBP4	−1.5	−1.8	−1.8	−2.5
609_f_at*	metallothionein 1B	MT1B	−1.4	−3.6	−1.3	−2.3
36130_f_at*	metallothionein 1E	MT1E	−1.4	−3.5	−1.2	−1.9
31622_f_at*	metallothionein 1F	MT1F	−1.5	−2.9	−1.4	−2.3
39594_f_at*	metallothionein 1H	MT1H	−1.5	−3.2	−1.4	−2.4
41530_at	acetyl-Coenzyme A acyltransferase 2 (mitochondrial 3-oxoacyl-Coenzyme A thiolase)	ACAA2	−1.1	−2	−1.1	−1.6
34050_at	acyl-CoA synthetase medium-chain family member 1	ACSM1	2.1	3.5	1.7	3.1
684_at	angiotensinogen (serpin peptidase inhibitor, clade A, member 8)	AGT	−1.9	−2.5	−3.3	−3.4
32747_at	aldehyde dehydrogenase 2 family (mitochondrial)	ALDH2	1.1	−1.6	1.1	−1.5
33756_at	amine oxidase, copper containing 3 (vascular adhesion protein 1)	AOC3	−1.1	−1.4	−1.2	−2.5
41306_at	amyloid beta (A4) precursor protein-binding, family A, member 2 binding protein	APBA2BP	1.2	1.7	1.4	1.5
287_at	activating transcription factor 3	ATF3	2.4	1.5	5.2	3.4
201_s_at	beta-2-microglobulin	B2M	1.1	−1.4	1.1	−1.4
2011_s_at	BCL2-interacting killer (apoptosis- inducing)	BIK	1.3	1.6	1.3	1.5
39409_at	complement component 1, r subcomponent	C1R	−1.2	−2.2	−1.4	−1.8
40496_at	complement component 1, s subcomponent	C1S	−1.1	−1.7	−1.2	−1.8
1943_at	cyclin A2	CCNA2	2	2.5	1.6	1.6
33950_g_at	corticotropin releasing hormone receptor 2	CRHR2	1.5	1.4	1.3	1.4
408_at	chemokine (C-X-C motif) ligand 1 (melanoma growth stimulating activity, alpha)	CXCL1	2.5	1.1	1.8	1.2
649_s_at	chemokine (C-X-C motif) receptor 4	CXCR4	3.3	2.6	3	2.8
38772_at	cysteine-rich, angiogenic inducer, 61	CYR61	1.7	−1.2	3.9	2.5
36643_at	discoidin domain receptor family, member 1	DDR1	1.7	1.6	1.6	1.5
33393_at	DEAD (Asp-Glu-Ala-As) box polypeptide 19B	DDX19B	−1.3	−1.7	−1.2	−1.5
32600_at	docking protein 4	DOK4	−1.4	−1.5	−1.4	−1.6
37827_r_at	dopey family member 2	DOPEY2	1.4	1.7	1.8	2.3
34823_at	dipeptidyl-peptidase 4 (CD26, adenosine deaminase complexing protein 2)	DPP4	1.8	2.4	2.9	2.7
36088_at	Down syndrome critical region gene 2	DSCR2	−2.4	−2.8	−1.3	−1.3
167_at	eukaryotic translation initiation factor 5	EIF5	−1.6	−2.1	−1.5	−1.8
1519_at	v-ets erythroblastosis virus E26 oncogene homolog 2 (avian)	ETS2	1.3	−1.5	−1.1	−1.9
36543_at	coagulation factor III (thromboplastin, tissue factor)	F3	2.6	1.9	2.7	2.7
1915_s_at	v-fos FBJ murine osteosarcoma viral oncogene homolog	FOS	2.2	1.2	5.8	3.9
36669_at	FBJ murine osteosarcoma viral oncogene homolog B	FOSB	1.5	1.2	5.7	4
39822_s_at	growth arrest and DNA-damage-inducible, beta	GADD45B	2.7	1.4	2.3	1.4
290_s_at	G protein-coupled receptor 3	GPR3	−1.1	−1.6	−1.2	−1.8
35127_at	histone cluster 1, H2ae	HIST1H2AE	1	1.4	1.2	1.7
31521_f_at	histone cluster 1, H4k	HIST1H4J	1	1.4	1	1.5
152_f_at	histone cluster 2, H4a	HIST2H4A	−1.6	−1.4	−1.5	−1.5
38833_at	major histocompatibility complex, class II, DP alpha 1	HLA-DPA1	3.3	2.5	1.4	1.1
38096_f_at	major histocompatibility complex, class II, DP beta 1	HLA-DPB1	2.8	1.8	1.5	1.1
36878_f_at	major histocompatibility complex, class II, DQ beta 1	HLA-DQB1	2.3	2.1	1.5	1.4
37039_at	major histocompatibility complex, class II, DR alpha	HLA-DRA	2.5	1.8	1.6	1.2
36617_at	inhibitor of DNA binding 1, dominant negative helix-loop-helix protein	ID1	−1.3	−2.2	−1	−1.5
676_g_at	interferon induced transmembrane protein 1 (9–27)	IFITM1	−1.4	−1.8	−1.9	−2.8
41745_at	interferon induced transmembrane protein 3 (1–8U)	IFITM3	−1.4	−1.6	−2.1	−3.2
37319_at	insulin-like growth factor binding protein 3	IGFBP3	1.8	−1.4	1.3	−1
36227_at	interleukin 7 receptor	IL7R	2.5	2.1	1.6	1.6
35372_r_at	interleukin 8	IL8	6.9	2.3	1.9	1.5
38545_at	inhibin, beta B (activin AB beta polypeptide)	INHBB	3	2.8	1.5	1.7
36355_at	involucrin	IVL	1.6	1.6	1.3	1.4
1895_at	jun oncogene	JUN	2.5	1.6	3	2.3
41483_s_at	jun D proto-oncogene	JUND	2.3	1.8	1.8	1.5
217_at	kallikrein-related peptidase 2	KLK2	1.8	2.1	5.7	6.5
35118_at	lecithin-cholesterol acyltransferase	LCAT	1.1	−1.8	1.1	−1.3
41710_at	hypothetical protein LOC54103	LOC54103	1.7	1.5	1.6	1.5
35926_s_at	lysozyme (renal amyloidosis)	LYZ	2.2	1.9	1.6	1.4
36711_at	v-maf musculoaponeurotic fibrosarcoma oncogene homolog F (avian)	MAFF	3	1.8	1.7	1.2
33146_at	myeloid cell leukemia sequence 1 (BCL2-related)	MCL1	2	1.4	1.5	1.2
33241_at	microfibrillar-associated protein 3-like	MFAP3L	−1.5	−1.9	−1.6	−1.8
668_s_at	matrix metallopeptidase 7 (matrilysin, uterine)	MMP7	2	1.2	4.1	2.8
870_f_at	metallothionein 3	MT3	−1.5	−2.8	−1.3	−2
36933_at	N-myc downstream regulated gene 1	NDRG1	1.6	1.9	1.5	1.5
37544_at	nuclear factor, interleukin 3 regulated	NFIL3	1.1	−1.3	1.2	−1.3
190_at	nuclear receptor subfamily 4, group A, member 3	NR4A3	2.2	1.6	1.6	1.2
31886_at	5’-nucleotidase, ecto (CD73)	NT5E	−1.3	−1.8	−1.2	−2.1
31733_at	purinergic receptor P2X, ligand-gated ion channel, 3	P2RX3	1.7	1.6	1.6	1.7
32210_at	phosphoglucomutase 1	PGM1	−1.2	−1.8	−1.1	−1.5
36980_at	proline-rich nuclear receptor coactivator 1	PNRC1	−1	−1.7	−1	−1.4
39366_at	protein phosphatase 1, regulatory (inhibitor) subunit 3C	PPP1R3C	−1.4	−1.9	−1.4	−2
36159_s_at	prion protein (p27–30) (Creutzfeldt-Jakob disease, Gerstmann-Strausler-Scheinker syndrome, fatal familial insomnia)	PRNP	1.2	−1.2	−1.1	−1.7
216_at	prostaglandin D2 synthase 21 kDa (brain)	PTGDS	2.3	1.8	1.4	−1.2
1069_at	prostaglandin-endoperoxide synthase 2 (prostaglandin G/H synthase and cyclooxygenase)	PTGS2	1.4	1	2.1	1.2
37701_at	regulator of G-protein signalling 2, 24 kDa	RGS2	3.1	2.3	1.2	−1.3
41471_at	S100 calcium binding protein A9	S100A9	−1.9	−4.1	−1.8	−2.4
33305_at	serpin peptidase inhibitor, clade B (ovalbumin), member 1	SERPINB1	−1.2	−1.6	−1	−1.6
36979_at	solute carrier family 2 (facilitated glucose transporter), member 3	SLC2A3	1.3	1.1	1.4	1.1
38797_at	solute carrier family 39 (zinc transporter), member 14	SLC39A14	−1.7	−2.9	−1.4	−1.9
38994_at	suppressor of cytokine signaling 2	SOCS2	2.2	1.4	1.4	1.1
34666_at	superoxide dismutase 2, mitochondrial	SOD2	−2.4	−3.2	−1.4	−1.8
38763_at	sorbitol dehydrogenase	SORD	1.7	1.4	2.4	2.5
38805_at	TGFB-induced factor homeobox 1	TGIF1	1.6	1.7	1.5	1.4
39411_at	TCDD-inducible poly (ADP-ribose) polymerase	TIPARP	2	1.6	1.8	1.3
1715_at	tumor necrosis factor (ligand) superfamily, member 10	TNFSF10	1.6	1.3	1.9	1.9
904_s_at	topoisomerase (DNA) II alpha 170 kDa	TOP2A	1.1	1.4	1.1	1.4
32793_at	T cell receptor beta variable 19	TRBC1	1.5	1.4	1.5	1.4
38469_at	tetraspanin 8	TSPAN8	1.7	2.2	1.6	1.6
40198_at	voltage-dependent anion channel 1	VDAC1	−1.5	−1.4	−1.3	−1.5
36909_at	WEE1 homolog (S. pombe)	WEE1	2	1.5	1.8	1.5
40448_at	zinc finger protein 36, C3H type, homolog (mouse)	ZFP36	2.9	1.5	2.6	1.7
32588_s_at	zinc finger protein 36, C3H type-like 2	ZFP36L2	2	1.2	1.4	1.1
1514_g_at			1.6	1.7	3.6	3.2
1662_r_at			1.9	2	3.6	4.1
40487_at	Transcribed locus		−1.2	−1.6	−1.1	−1.5

## Figures and Tables

**Table 1:** Overview of data sets used in batch I and batch II analyses.

**Batch I Analysis**				
	**Organ donor (N)**	**Adjacent to tumor (A)**	**Tumor (T)**	**Total**

Liver	21	30	43	94
Prostate	23	59	66	148

**Batch II Analysis**				
	**Organ donor (N)**	**Tumor (T)**		**Total**

Liver	21	43		64
Prostate	23	66		89
Lung	17	134		151
Bladder	5	57		62

**Table 2:** Prediction performance indexes (PPI) in batch I analysis. Pairwise two-group comparisons (N vs. T, N vs. A and A vs. T) are performed.

**Liver vs. Prostate (Normal vs. Tumor)**				
	**liv→liv**	**pro→liv**	**pro→pro**	**liv→pro**

All genes	96.5%	66.3%	93.9%	47.4%
Common signature	96.5%	93.0%	98.8%	96.3%

**Liver vs. Prostate (Normal vs. Adjacent)**				

All genes	92.6%	77.9%	96.6%	54.6%
Common signature	98.2%	96.0%	98.3%	96.6%

**Liver vs. Prostate (Adjacent vs. Tumor)**				
All genes	79.9%	51.9%	71.4%	55.7%
Common signature	75.6%	74.7%	66.7%	65.1%

**Table 3:** Prediction performance indexes (PPI) in batch II analysis. The values shaded in grey are summarized in Table 4.

**Liver vs. Prostate**				
	**liv→liv**	**pro→liv**	**pro→pro**	**liv→pro**

All genes	96.51%	66.28%	93.94%	47.36%
Common signature	97.67%	97.67%	95.55%	94.14%
**Liver vs. Lung**				
	**liv**→**liv**	**lun**→**liv**	**lun**→**lun**	**liv**→**lun**

All genes	96.51%	56.98%	90.72%	45.32%
Common signature	95.23%	93.02%	95.94%	94.72%
**Lung vs. Prostate**				
	**lun**→**lun**	**pro**→**lun**	**pro**→**pro**	**lun**→**pro**
All genes	90.72%	69.03%	93.94%	62.88%
Common signature	94.82%	94.45%	79.61%	72.76%
**Liver vs. Bladder**				
	**liv**→**liv**	**bla**→**liv**	**bla**→**bla**	**liv**→**bla**

All genes	96.51%	62.79%	88.60%	49.65%
Common signature	91.74%	91.86%	98.25%	98.25%
**Prostate vs. Bladder**				
	**pro**→**pro**	**bla**→**pro**	**bla**→**bla**	**pro**→**bla**

All genes	93.94%	36.30%	88.60%	42.63%
Common signature	92.92%	86.86%	97.81%	88.25%
**Lung vs. Bladder**				
	**lun**→**lun**	**bla**→**lun**	**bla**→**bla**	**lun**→**bla**

All genes	90.72%	51.87%	88.60%	50.88%
Common signature	89.38%	85.91%	97.37%	85.61%

**Table 4:** PPI summary of within-cancer-type and inter-cancer-type predictions in batch II analysis.

		Test data			
		Liver	Prostate	Lung	Bladder
**Training data**	Liver	96.5% (69)*	94.1% (225)+	94.7% (119)+	98.3% (53)+
	Prostate	97.7% (225)+	93.9% (55)*	94.5% (288)+	88.3% (10)+
	Lung	93.0% (119)+	72.8% (288)+	90.7% (57)*	85.6% (19)+
	Bladder	91.9% (53)+	86.9% (10)+	85.9% (19)+	88.6% (135)*

* All genes are used in the within-cancer-type prediction to allow PAM for automatic predictive gene selection. Numbers of genes used in PAM are shown in parentheses.

+ In all inter-cancer-type predictions, only common signature genes are used in PAM and PAM does not perform further gene selection. The numbers of genes appeared more than 70% of leave-one-out cross validations are shown in the parentheses (i.e. liv-pro-MBs, liv-lun-MBs and pro-lun-MBs).

**Table 5:** The 44 batchII-MBs overlapped by pair-wise comparisons of liver, prostate and lung data sets (liv-pro-MB, liv-lun-MB, pro-lun-MB). The first 12 genes with asterisk overlapped batchI-MBs. The signed mean fold change shows mean fold change of tumor versus normal when positive (up-regulation) and normal versus tumor when negative (down-regulation).

Probe set ID	Gene title	Gene symbol	Signed mean fold change		
			Liver	Prostate	Lung
39597_at*	actin binding LIM protein family, member 3	ABLIM3	−2.1	−1.6	−2
37599_at*	aldehyde oxidase 1	AOX1	−2.8	−2.6	−1.5
34736_at*	cyclin B1	CCNB1	2.3	1.8	1.7
37302_at*	centromere protein F, 350/400 ka (mitosin)	CENPF	1.9	1.4	1.4
37203_at*	carboxylesterase 1 (monocyte/macrophage serine esterase 1)	CES1	−1.8	−1.7	−3
32168_s_at*	Down syndrome critical region gene 1	DSCR1	−2	−1.6	−1.8
34311_at*	glutaredoxin (thioltransferase)	GLRX	−2.5	−1.7	−1.5
1737_s_at*	insulin-like growth factor binding protein 4	IGFBP4	−1.8	−2.5	−1.6
609_f_at*	metallothionein 1B	MT1B	−3.6	−2.3	−1.5
36130_f_at*	metallothionein 1E	MT1E	−3.5	−1.9	−1.8
31622_f_at*	metallothionein 1F	MT1F	−2.9	−2.3	−1.8
39594_f_at*	metallothionein 1H	MT1H	−3.2	−2.4	−1.7
35699_at	BUB1 budding uninhibited by benzimidazoles 1 homolog beta (yeast)	BUB1B	1.5	1.4	1.3
38796_at	complement component 1, q subcomponent, B chain	C1QB	−2.4	−1.4	−2.3
35276_at	claudin 4	CLDN4	1.4	2.4	1.4
36668_at	cytochrome b5 reductase 3	CYB5R3	−1.4	−1.4	−1.5
33295_at	Duffy blood group, chemokine receptor	DARC	−1.7	−2.9	−1.4
41225_at	dual specificity phosphatase 3 (vaccinia virus phosphatase VH1 related)	DUSP3	−1.4	−1.4	−1.5
38052_at	coagulation factor XIII, A1 polypeptide	F13A1	−1.7	−2.1	−1.5
37743_at	fasciculation and elongation protein zeta 1 (zygin I)	FEZ1	−1.5	−1.6	−2
38326_at	G0/G1switch 2	G0S2	−3.1	−2.2	−1.7
1597_at	growth arrest-specific 6	GAS6	−1.6	−2	−1.7
411_i_at	interferon induced transmembrane protein 2 (1–8D)	IFITM2	−1.6	−2	−1.4
37484_at	integrin, alpha 1	ITGA1	−1.6	−1.4	−1.3
38116_at	KIAA0101	KIAA0101	2.2	1.6	1.3
37883_i_at	Hypothetical gene supported by AK096951	LOC400879	1.5	1.7	1.4
242_at	microtubule-associated protein 4	MAP4	−1.4	−1.6	−1.4
31623_f_at	metallothionein 1A	MT1A	−3.5	−2.6	−1.4
39081_at	metallothionein 2A	MT2A	−2	−2.5	−2.1
37736_at	protein-L-isoaspartate (D-aspartate)	PCMT1	−1.6	−1.3	−1.3
35752_s_at	O-methyltransferase protein S (alpha)	PROS1	−2.2	−1.7	−2
34163_g_at	RNA binding protein with multiple splicing	RBPMS	−1.5	−2.4	−1.4
34887_at	Radixin	RDX	−1.5	−1.4	−1.7
39150_at	ring finger protein 11	RNF11	−1.6	−1.4	−1.3
41096_at	S100 calcium binding protein A8	S100A8	−3.6	−2.3	−3.3
33443_at	serine incorporator 1	SERINC1	−1.8	−1.5	−1.6
39775_at	serpin peptidase inhibitor, clade G (C1 inhibitor), member 1, (angioedema, hereditary)	SERPING1	−1.6	−2.2	−2
1798_at	solute carrier family 39 (zinc transporter), member 6	SLC39A6	1.4	1.6	1.4
33131_at	SRY (sex determining region Y)-box 4	SOX4	2.5	1.8	1.8
40419_at	stomatin	STOM	−1.5	−1.8	−2
1897_at	transforming growth factor, beta receptor III	TGFBR3	−1.4	−1.7	−2.5
38404_at	transglutaminase 2 (C polypeptide, protein-glutamine-gamma-glutamyltransferase)	TGM2	−2.9	−1.8	−1.8
40145_at	topoisomerase (DNA) II alpha 170 kDa	TOP2A	1.6	1.7	2
35720_at	WD repeat domain 47	WDR47	−2.4	−1.5	−1.3
